# Dynamic neuromuscular stabilization, balance, and conventional training for chronic ankle instability in amateur athletes: a randomised controlled trial

**DOI:** 10.1186/s13102-025-01319-8

**Published:** 2025-10-01

**Authors:** Sevval Yesilkir, Gizem Ergezen Sahin

**Affiliations:** 1 Department of Physical Therapy and Rehabilitation, Graduate School of Health Sciences, Istanbul Medipol University, Istanbul, 34810 Turkey; 2 Department of Physical Therapy and Rehabilitation, Faculty of Health Sciences, Istanbul Medipol University, Istanbul, 34810 Turkey; 3 Department of Physical Therapy and Rehabilitation, Istanbul Medipol University School of Health Science, Atatürk St. 40/16, Istanbul, 34815 Turkey

**Keywords:** Chronic ankle instability, Amateur athlete, Sports rehabilitation, Dynamic neuromuscular stabilization, Balance training, Vestibular training, Motor control

## Abstract

**Objectives:**

The aim of this study was to compare the effects of three distinct rehabilitation approaches, namely Dynamic Neuromuscular Stabilization (DNS) training, balance training and conventional physiotherapy, on neuromuscular control and functional performance in amateur athletes with chronic ankle instability (CAI).

**Trial design and framework:**

A single-blind, parallel-group randomized controlled trial with a superiority framework was conducted.

**Methods:**

Amateur athletes with chronic ankle instability were recruited from sports clinics in Istanbul. A total of 40 participants (18 males, 22 females) from football, gymnastics, rowing, taekwondo, tennis, volleyball, and swimming were randomly assigned (1:1:1) using a computer-generated permuted block design to DNS training (DNSG) (*n* = 13), balance training (BTG) (*n* = 14), or conventional training (CTG) (*n* = 13). Interventions were delivered three times per week for six weeks. DNS involved breathing-centered stabilization exercises; balance training included structured proprioceptive and vestibular tasks; and conventional training used standard strength and posture control exercises. Outcome assessors were blinded, while participants and therapist were not.

**Results:**

A total of thirty-six participants completed the trial, with the following group distributions: DNSG (*n* = 12), BTG (*n* = 13), and CTG (*n* = 11). Both DNSG and BTG showed significantly greater improvements than the CTG across all outcome measures (*p* < 0.001), with large effect sizes (FAAM-ADL η²=0.97; YBT-A η²=0.92; SHT η²=0.95). DNS was significant in postural control (*p* = 0.01), while balance training showed greater improvements in reaction time (RT) (*p* = 0.02). No significant between-group differences were observed in CAIT scores, though DNSG and BTG demonstrated large within-group improvements. No serious harms or adverse events were reported in any of the groups.

**Conclusions:**

DNS and balance training are more effective than conventional physiotherapy in improving neuromuscular function, postural control, and performance in amateur athletes with CAI. These interventions offer complementary benefits and may be integrated into targeted rehabilitation protocols to optimize outcomes and support return-to-sport strategies.

**Trial registration:**

Clinical trial approval was obtained at https://www.clinicaltrials.gov/, and the registration status was made publicly available with the number of NCT06296537 on February 29, 2024. The registry record can be accessed at https://clinicaltrials.gov/study/NCT06296537. As of the time of writing, the results have not yet been published on the registry.

**Supplementary Information:**

The online version contains supplementary material available at 10.1186/s13102-025-01319-8.

## Introduction

Chronic ankle instability (CAI) results from recurrent lateral ankle sprains, causing persistent pain, instability, sensations of the ankle “giving way”, structural changes, and adaptations in the sensorimotor and vestibular systems. It represents a significant global musculoskeletal health concern with implications that extend far beyond the athletic population [1–3]. The long-term consequences of CAI include not only sensorimotor and vestibular system dysfunction, but also early-onset post-traumatic osteoarthritis of the ankle, reduced physical activity levels, and decreased quality of life, even in young individuals [4, 5]. Epidemiological data indicate that the prevalence of CAI varies by age and activity level, with rates up to 29% among high school students, 23.4% in high school and college athletes and 20% in adolescent athletes [6–8]. Importantly, this condition is frequently underdiagnosed or misperceived as benign, leading to insufficient rehabilitation and high recurrence rates [6, 9].

Ankle deformity recovery, particularly in cases of CAI, requires a multifaceted approach that integrates conservative treatments, including physical therapy, external support, and psychological support [10, 11]. Physical therapy interventions commonly integrate strength exercises, proprioceptive training, and range-of-motion exercises with manual therapy techniques [3, 10, 12, 13]. However, conventional training methods may not fully address the dynamic and complex nature of ankle instability, and may not sufficiently restore neuromuscular control or prevent recurrence of instability. Therefore, the International Ankle Consortium advocates for evidence-informed rehabilitation protocols that address both sensorimotor and arthrokinematic impairments, to reduce recurrence risk and optimize long-term outcomes​ [5, 14, 15].

Balance and vestibular training has been shown to enhance proprioception, postural stability, and neuromuscular control factors that are vital for restoring ankle function and reducing the risk of reinjury in individuals with CAI [16, 17]. However, one emerging approach that may offer even broader benefits is Dynamic Neuromuscular Stabilization (DNS). Dynamic Neuromuscular Stabilization is based on developmental kinesiology principles, aiming to correct dysfunctional movement strategies and restore efficient motor control by replicating optimal early motor patterns [18, 19]. At its core, DNS works by activating the body’s intrinsic stabilization system, stimulating the brain’s natural motor control pathways. This is achieved through focused breathing techniques, core activation, and posture-specific exercises that help regulate intra-abdominal pressure (IAP) and optimize spinal and joint alignment [20]. DNS highlights the crucial role of diaphragmatic breathing in creating a stable foundation for efficient limb movement, allowing for improved force transfer, balance, and coordination during dynamic tasks [21].

The DNS approach has demonstrated effectiveness in treating conditions such as chronic musculoskeletal pain [20, 22], and sports rehabilitation [18, 19, 23]. Functional stabilization training, which shares basic principles with DNS, has been shown to improve balance and motor coordination in individuals with neuromusculoskeletal disorders such as Charcot-Marie-Tooth disease [24]. Similarly, DNS training in collegiate athletes has led to significant improvements in dynamic balance as measured by the Y Balance Test, a validated assessment of ankle function [25]. These findings provide indirect but important evidence that DNS may improve postural control and functional stability, which are key rehabilitation goals in individuals with CAI. However, further research is required to investigate its specific effects on ankle sensorimotor control.

Despite the benefits of traditional approaches, conventional training alone may fall short in addressing the complex neuromuscular adaptations and sensorimotor deficits associated with CAI [26]. This underscores the need for innovative rehabilitation strategies like DNS, which offer a more integrated, holistic framework that may not only improve joint stability but also support long-term injury prevention and optimal athletic performance [15, 21].

To our knowledge, no randomized controlled trial has directly compared the effects of DNS and structured balance training on functional outcomes in amateur athletes with CAI. Therefore, the aim of this study is to compare the effects of DNS-based rehabilitation, balance training, and conventional training on key neuromuscular control and functional performance outcomes in amateur athletes with CAI. The current trial aims to fill this gap by providing robust comparative evidence that is expected to inform clinical practice and rehabilitation guidelines worldwide. It was hypothesized that DNS and balance training would lead to greater improvements in neuromuscular control and functional performance compared to conventional physiotherapy in amateur athletes with chronic ankle instability.

## Methods

Patients or members of the public were not involved in the design, conduct, reporting, or dissemination of this research.

### Study design

This study was designed as a single-blind, parallel-group, randomized controlled trial with a superiority framework. The unit of randomization was the individual participant, and participants were randomly assigned to one of three intervention groups with an allocation ratio of 1:1:1. The aim was to compare the effectiveness of DNS, balance training, and conventional training in improving functional outcomes in amateur athletes with chronic ankle instability.

This study was conducted in accordance with CONSORT guidelines [27]. Prior to the initiation of the study, the protocol was registered on https://www.clinicaltrials.gov/, and registration status was made publicly available under the identifier NCT06296537. The study was granted ethical approval by the Istanbul Medipol University Non-Invasive Clinical Research Ethics Committee on February 15, 2024, under decision number 181/ E-10840098-202.3.02-1434.

#### Settings

The trial was conducted in clinical and academic physiotherapy settings in Istanbul, Turkey. Participants were assessed and treated at the Istanbul Medipol University Hospital, Department of Physiotherapy and Rehabilitation, and the Söğütlüçeşme Birlik Sports Club Physiotherapy and Rehabilitation Clinic. These locations provided appropriate facilities for delivering supervised exercise interventions and conducting functional assessments in amateur athletes with chronic ankle instability.

### Participants

Participants were recruited through referrals from physicians at the Departments of Sports Medicine and Orthopedics, Söğütlüçeşme Birlik Sports Club, Destanların Dansı Dance Club, and physiotherapists at the Istanbul Medipol University Physiotherapy and Rehabilitation Clinic. All eligible participants were screened according to the inclusion criteria after referral, and informed consent was provided before enrollment.

A diagnosis of CAI was required as confirmed by a sports medicine physician or orthopedist according to the International Ankle Consortium criteria [3, 28]. The athletes selected for the study had a score 25 or lower on the CAIT and met al.l other relevant inclusion criteria. The inclusion criteria for this study were as follows: nonsmokers, to have experienced at least two episodes of ankle instability in the past six months, and to have experienced at least one acute ankle inversion sprain, which caused pain, swelling, and functional impairment at least 12 months prior to the study [29, 30]. To ensure similar activity levels, defined as individuals engaging in recreational or competitive sports for at least 3 h per week. Participants with bilateral CAI were referred to the hospital’s physiotherapy department for necessary rehabilitation and were excluded from the study. Exclusion criteria included: mental health disorders, neurological disorders, or dysfunctions of the cerebellum, vestibular system, cochlea, or inner ear [31]; previous surgery on musculoskeletal structures; chronic musculoskeletal disorders; lumbar herniation; severe acute lower extremity injuries; or head trauma within the past six months [32, 33]. Additionally, participants who missed more than two of the 18 sessions during the six-week study period were excluded from the study after being provided with home exercises.

#### Intervention

Participants were randomized into three groups: the DNSG, the BTG, and CTG. All groups participated in supervised physiotherapy sessions three times per week for six weeks, with each session lasting approximately 45–60 min. Interventions were delivered by a certified physiotherapist with over three years of experience and expertise in DNS and Neuroathletic Performance. The DNSG received breathing-centered DNS positions, tailored to each participant’s limitations. The exercises incorporated diaphragmatic breathing and positions such as Baby Rock, prone, oblique sitting, tripod, high kneeling, hanging (midstance and push-up), bear, and squat (Appendix A). Breathing exercises, designed to target diaphragm descent, rib expansion, and thoracic mobility were combined with positions. Additionally, foot activation exercises were performed by participants in DNS positions, with a piece of paper placed under the toes, heel, and both the medial and lateral arches of the foot. While the researcher randomly drew a piece of paper, the participants were instructed to prevent the paper from being drawn. Sessions included progressive resistance using elastic bands, sandbags, and balls, and incorporated barefoot training and foot activation drills to stimulate sensorimotor integration [29, 33].

BTG participants received a structured balance and vestibular training program consisting of static and dynamic balance tasks with progressive load adjustments and the use of resistance materials as needed [32, 34, 35] (Appendix B).

The CTG received conventional physiotherapy, including ROM, strengthening, and postural control exercises, also performed barefoot and progressed in volume and resistance (Appendix C). For all interventions, progression was adjusted based on participants’ ability to perform the prescribed exercises with correct technique, absence of pain, and completion of the prescribed repetitions or duration without excessive fatigue. When these criteria were met for two consecutive sessions, exercise difficulty was increased by modifying parameters such as base of support, surface stability, resistance, range of motion, or cognitive load, depending on the intervention type. Educational information and home exercise guidance were also provided to all groups. Specifically, each participant received a brief educational session at the beginning of the program, which included information on ankle anatomy, the mechanisms of chronic ankle instability, injury prevention strategies, and the importance of adherence to rehabilitation exercises.For home exercise guidance, participants were given illustrated instructions for the exercises. These exercises were selected to complement supervised sessions and maintain training effects between visits. Participants were instructed to perform the home exercises three times per week, with each session lasting approximately 15–20 min. Compliance was encouraged by weekly verbal follow-up during supervised sessions, and participants were asked to report any pain, discomfort, or difficulties encountered.

Adherence was monitored by attendance tracking; participants missing more than two sessions were excluded. No additional concomitant treatments were permitted during the study period. Fidelity was ensured by standardizing protocols across sessions and assigning a single trained therapist to each intervention arm. Intervention details and progression guidelines were documented in an internal exercise manual.

Safety and tolerability were assessed at the end of each intervention session by asking participants whether they experienced any pain, discomfort, or adverse effects related to the intervention.

#### Outcomes

At the commencement of the study, participants completed a demographic information form to collect essential baseline data, including age, height, weight, body mass index, and sport discipline. Assessments were conducted at three specific time points: the baseline (pre-test), following six weeks of training (post-test), and at 12 weeks (follow-up test). A comprehensive set of parameters was systematically evaluated at each time point to assess participants’ progress and overall outcomes. The primary outcomes of the trial were dynamic balance and functional ankle instability, measured respectively by the YBT and the CAIT. The secondary outcomes included self-reported functionality (Foot and Ankle Ability Measure–FAAM), static balance (Balance Error Scoring System–BESS), stabilization (Foot Lift Test–FLT), reaction time (BlazePod™ Reaction Time Test-RT), and functional performance (Side Hop Test–SHT). These outcomes were selected based on prior literature as reliable, validated, and responsive tools for assessing neuromuscular and functional recovery in individuals with chronic ankle instability. They align with recommendations from the International Ankle Consortium but are not part of a formal core outcome set [5] All analyses were conducted by a blinded physiotherapist (G.E.S.) who was not involved delivering the interventions from specialized research groups to minimize bias. The interventions were applied by another physiotherapist (S.Y.).

The YBT, an advanced adaptation of the Star Excursion Balance Test, was utilized to evaluate dynamic balance. Participants stood on their injured leg while reaching in the anterior, posteromedial and posterolateral directions with the non-injured leg, positioning their hands on their hips to ensure balance. YBT reach distances were normalized to leg length, measured as the distance from the anterior superior iliac spine to the distal tip of the medial malleolus in a supine position. Invalid trials were repeated. Following the completion of three practice trials, the mean of three valid reach distances for each direction was noted [36, 37]. The CAIT is designed to assess functional instability of the ankle, consisting of 9 items with a maximum possible score of 30. A score of 25 or higher is indicative of a lower risk of instability, whereas a lower score of 25 indicates an increased risk [38]. The CAIT has been validated and is considered reliable in Turkish [39].

The FAAM is a tool used to assess self-reported functional abilities in individuals with foot, ankle, or leg conditions. It contains two subscales: one for activities of daily living and another for sports. Each question is scored from 4 (no difficulty) to 0 (inability to perform). The total score, expressed as a percentage (0-100%), reflects the individual’s level of functionality, with higher percentages indicating better functional capacity [40, 41]. The FAAM has been shown to be both valid and reliable in Turkish [42].

The FLT was utilized to evaluate stability. Participants were instructed to stand on their affected leg, eyes closed and hands on hips, while maintaining an upright posture for 30 s. Errors were defined as any instance of lifting the foot off the ground or the opposite foot touching the ground. Following a single practice trial, participants completed three test trials, with a 30-second rest interval between each. The trial exhibiting the highest number of errors was chosen for analysis [43].

The BESS was used to assess static balance, consisting of six 20-second stances: double-leg, single-leg, and tandem positions on both firm and foam surfaces, with eyes closed. Errors such as opening the eyes, lifting the forefoot or heel, stepping, stumbling, falling, removing hands from the iliac crests, hip abduction/flexion greater than 30°, or remaining out of the test position for more than 5 s were recorded, with simultaneous errors counted as a single error. The maximum error score for each stance was 10, yielding a total possible range of 0–60, with lower scores indicating better static balance. For analysis, the total error score (BESS-Total) was calculated as the sum of errors across all stances, and scoring was performed manually by the blinded outcome assessor [44, 45].

BlazePod™ technology, utilized to track performance improvements and evaluate the impact of a training intervention on RT, is considered suitable for practical implementation in physically active young adults, with a moderate ICC and an excellent CV observed [46]. The test was conducted using a 36-inch-long, 12-inch-wide rectangle area, with four Blazepods placed at each corner. The participant, standing on one foot at the center of the rectangle, was tasked with responding to the lights as quickly as possible for 30 s. The test was repeated three trials, and the highest number of responses was recorded [47].

The SHT is recognized as a highly valid performance test for identifying patients with CAI [48]. During the test, participants are instructed to complete 10 quick round trips on a single foot, alternating between medially and laterally, across two parallel lines placed 30 cm apart. The duration taken to complete the trial is recorded. The test was administered twice on the affected foot, with a 60-second rest period between trials. The trial with the shortest completion time was selected for analysis [49].

Participants were instructed to report any discomfort, pain, or injury experienced during or after the exercise sessions throughout the 6-week intervention period and at the 12-week follow-up. Reports of potential harms were collected verbally at each session by the treating physiotherapist and documented in case report forms.

#### Sample size

The sample size for this study was determined using G-Power^®^ software (version 3.1.9, Universität Düsseldorf, Germany), a repeated measures ANOVA (within-between interaction) model was selected for the analysis [50]. The primary hypothesis tested was “the effect of Y Balance Test-Anterior (YBT-A) measurement values, considering both time (3 measurements) and group (3 groups).” In the absence of a comparable study in the literature, the effect size was assumed to be the lower bound of the medium effect size (0.27). Furthermore, the Type I error rate was set at α = 0.05 (95% confidence interval), and the desired statistical power was set at 1-β = 0.95. Based on these parameters, the required sample size for the analysis was calculated to be 36. To account for potential dropouts or anticipated at 10–15% of non-adherence, the total sample size was increased to 40 participants, with a target of approximately 12–14 per group. No interim analyses were planned or conducted. As such, no formal stopping guidelines, adaptive sample size re-estimation strategies, or involvement of a Data Monitoring Committee (DMC) were employed. The study proceeded to full recruitment and completion as originally designed, with all data analyzed after the final follow-up assessments.

### Randomization

This trial used restricted randomisation with a permuted block design to ensure balanced group sizes throughout recruitment. A computerized random number generator was used via *Randomizer.org* to generate the allocation sequence. Participants were randomly assigned in a 1:1:1 ratio to one of three groups: DNSG, BTG, or CTG. The block size was concealed from the researchers responsible for participant enrollment to maintain allocation concealment and prevent prediction of upcoming assignments. No stratification or minimisation techniques were applied.

The random allocation sequence was generated and securely stored by an independent researcher who was not involved in enrollment, outcome assessment, or intervention delivery. Only this individual had access to the full sequence. Participant enrollment was conducted by physiotherapists and research staff who screened for eligibility and obtained informed consent. Following consent, group assignments were implemented using sequentially numbered, opaque, sealed envelopes (SNOSE) prepared in advance and stored in a locked cabinet. Enrolling staff had no access to the randomization list at any point, thereby ensuring strict allocation concealment and minimizing selection bias.

All participants were fully informed about the study’s purpose, procedures, potential benefits, and risks. Written informed consent was obtained prior to inclusion in the trial.

#### Blinding

This was a single-blind trial, in which the outcome assessor was blinded to group allocation to reduce detection bias. Due to the nature of the interventions, participants and treating physiotherapist were not blinded. The blinded assessor, a physiotherapist independent of the intervention process, conducted all pre-test, post-test, and follow-up evaluations and had no access to group assignment records. Participants were instructed not to reveal their group to the assessor. Additionally, the data analyst was blinded during statistical processing, as datasets were coded anonymously (Group A, B, C). No placebo or dummy interventions were used, and no formal blinding validation procedures were performed. No cases of unintentional unblinding occurred during the trial.

### Data analysis

All data were recorded and analyzed using SPSS (statistical package for the social sciences) for Windows 22. Analyses were conducted on an intention-to-treat basis, including all randomized participants who completed at least one post-intervention assessment. Preliminary assumptions regarding participant’s data were assessed using the Shapiro Wilk test to evaluate the normality, along with an examination of skewness and kurtosis values, which provided additional support for confirming the normal distribution. Based on the results of the Shapiro–Wilk test, variables with *p* > 0.05 and kurtosis and skewness values within the range of ± 2.0 were considered to exhibit no significant deviation from normality [51, 52]. Homogeneity of variances was assessed using Levene’s test before conducting ANOVA procedures. Continuous data were reported as mean ± standard deviation, along with median, while categorical data were expressed as percentages.

The primary outcomes (YBT and CAIT scores) and secondary outcomes (FAAM, BESS, FLT, BlazePod™ RT, and Side Hop Test) were assessed using mixed-design repeated measures ANOVA to evaluate differences between groups (DNSG, BTG, CTG) and across time points (pre-test, post-test, follow-up). When significant main or interaction effects were observed, Bonferroni post-hoc tests were applied to determine specific group differences. One-way ANOVA was used for comparing baseline characteristics. For categorical variables, the Chi-square test or Fisher’s exact test was used as appropriate. A significance level of 0.05 was adopted as the criterion for determining the statistical significance of the results. The confidence interval for the study was determined as 95% and the significance threshold as 5%.

## Results

**Fig. 1:**
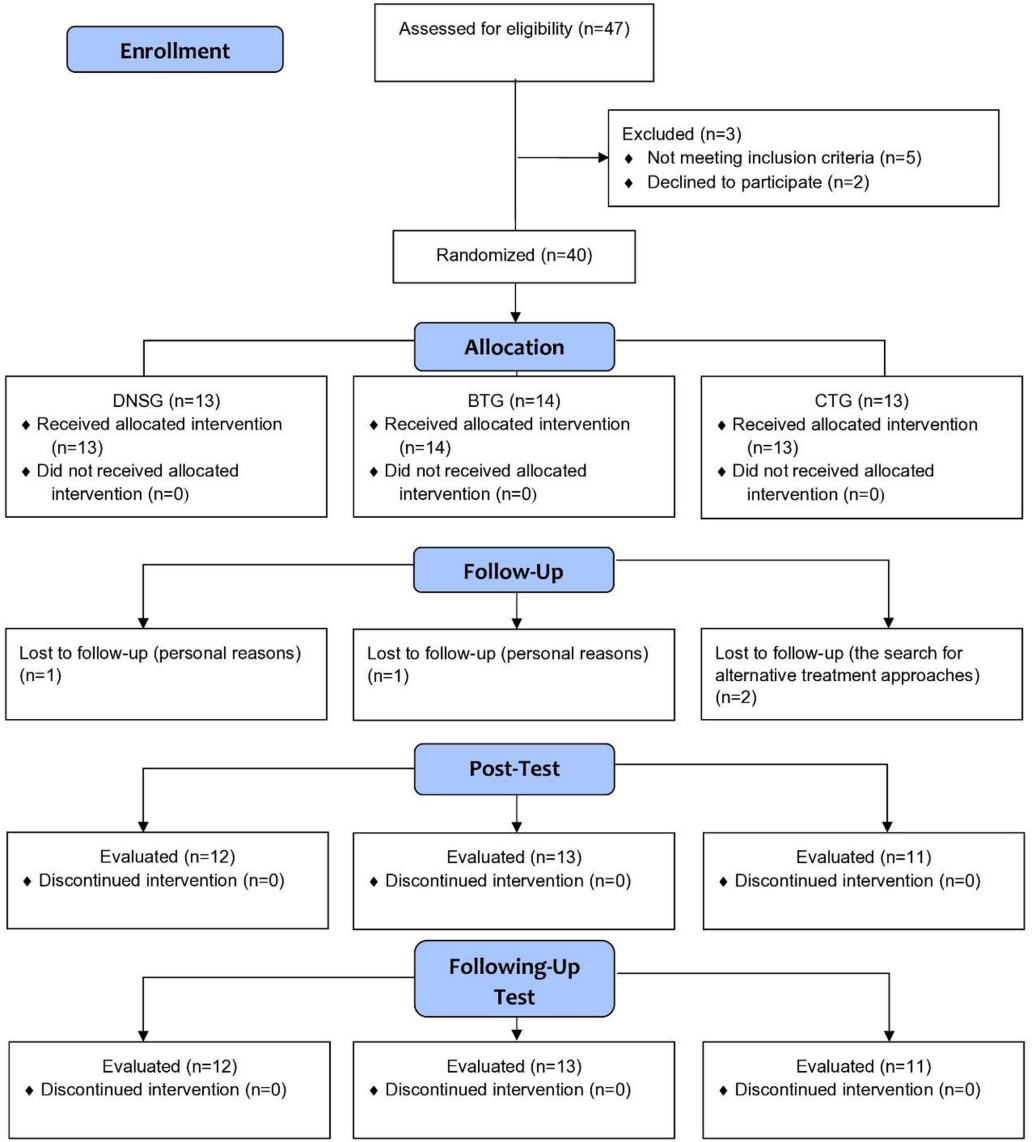
CONSORT flow diagram

A total of 47 patients were initially evaluated for eligibility to participate in the study, A total of 40 participants were randomized into three groups: DNSG, *n* = 13, BTG, *n* = 14, and CTG, *n* = 13. Of these, 36 participants completed the intervention and follow-up assessments, while 4 participants (one from DNSG, one from BTG, and two from CTG) withdrew before completing the post-test. Therefore, the number of participants who received the intended intervention was 12 in DNSG, 13 in BTG, and 11 in CTG. All participants who completed the post-test were included in the analysis for the primary outcomes (YBT and CAIT), in accordance with the intention-to-treat approach. A graphical representation of participant flow is provided in Fig. 1, following the CONSORT diagram (Fig. 1).

Participant recruitment began on April 1, 2024, and was completed on November 11, 2024. The final follow-up assessment was conducted on December 16, 2024, marking the end of the study period. Each participant was followed for a total duration of 12 weeks, consisting of a 6-week intervention period and a 6-week follow-up phase. The follow-up duration was uniform across participants, with a minimum and maximum follow-up of 12 weeks.

At the beginning of the study, no significant differences in demographic characteristics were observed between the groups, as presented in Table 1.

**Table 1:** Baseline participant characteristics for the dynamic neuromuscular stabilization, balance and conventional training groups at baseline

Variable	Group	GROUPS			Test
		DNSG (*N* = 13)	BTG (*N* = 14)	CTG (*N* = 13)	
		*n* (%)	*n* (%)	*n* (%)	
**Sex**	Female	8 (61.5%)	5 (35.7%)	8 (61.5%)	X^2^:2.43; p:0.30
	Male	5 (38.5%)	9 (64.3%)	5 (38.5%)	
**Sport disciplines**	Football	5 (38.5%)	6 (42.9%)	7 (53.9%)	X^2^:0.94; p:0.99
	Gymnastics	1 (7.7%)	1 (7.1%)	1 (7.7%)	
	Rowing	1 (7.7%)	1 (7.1%)	1 (7.7%)	
	Taekwondo	1 (7.7%)	2 (14.3%)	1 (7.7%)	
	Tennis	1 (7.7%)	1 (7.1%)	1 (7.7%)	
	Volleyball	1 (7.7%)	1 (7.1%)	1 (7.7%)	
	Swimming	3 (23.1%)	3 (21.4%)	2 (15.4%)	
**Chronic illnesses**	Yes	1 (7.7%)	-	-	X^2^:1.72; p:1.00
	No	12 (92.3%)	14 (100%)	13 (31%)	
**Past illnesses**,** surgeries**	Yes	-	2 (14.3%)	-	X^2^:4.81; p:0.15
	No	13 (100%)	12 (85.7%)	13 (30%)	
**BMI (kg/m²)**	X̅±SD	23.18 ± 2.78	21.71 ± 1.55	21.82 ± 3.69	F:0.63; p:0.54
**Age (Years)**	X̅±SD	22.54 ± 2.67	21.64 ± 2.59	21.54 ± 2.85	F:0.65; p:0.53
**FAAM-DLA (%)**	X̅±SD	71.09 ± 9.33	75.64 ± 7.74	70.92 ± 7.26	F:1.475; p:0.24
**FAAM-Sport (%)**	X̅±SD	64.86 ± 7.71	68.00 ± 7.40	65.22 ± 6.82	F:0.748; p:0.48
**CAIT (Score)**	X̅±SD	15.46 ± 2.99	16.93 ± 3.56	15.31 ± 3.28	F:1.008; p:0.38
**FLT**	X̅±SD	7.23 ± 1.30	7.83 ± 1.11	8.50 ± 1.35	F:2.893; p:0.07
**BESS-Total**	X̅±SD	19.85 ± 2.58	20.36 ± 3.05	20.08 ± 3.23	F:0.101; p:0.91
**YBT-A**	X̅±SD	67.46 ± 3.20	67.36 ± 3.25	65.77 ± 4.15	F:0.936; p:0.40
**YBT-PL**	X̅±SD	87.62 ± 2.53	88.71 ± 3.79	86.46 ± 3.33	F:1.594; p:0.22
**YBT-PM**	X̅±SD	90.92 ± 2.33	91.79 ± 3.33	90.30 ± 3.28	F:0.817; p:0.45
**BlazePod™ RT**	X̅±SD	22.85 ± 3.02	23.00 ± 1.96	22.85 ± 2.16	F:0.019; p:0.98
**SHT**	X̅±SD	10.71 ± 2.38	9.23 ± 2.46	9.02 ± 1.19	F:2.504; p:0.10

F: Independent sample t-test; X^2^: Chi square test; X̅±SD: Mean ± Standard deviation; BMI: Body mass index

**Fig. 2:**
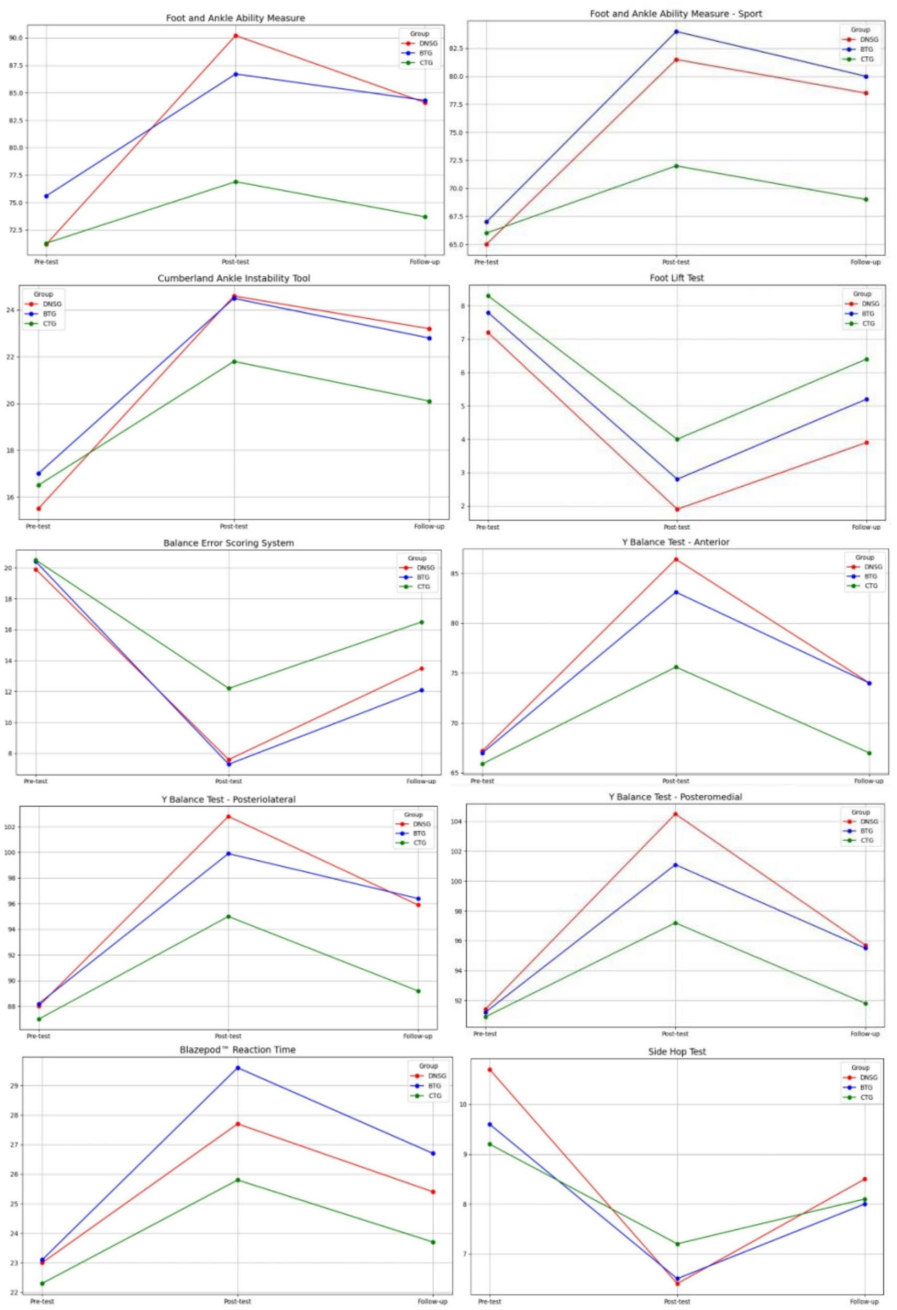
Change of the evaluation parameters throughout time in dynamic neuromuscular stabilization, balance and conventional training groups based on the repeated-measure analysis of variance (estimated marginal means are shown). Abbreviations: DNSG, dynamic neuromuscular stabilization training group; balance training group, BTG; CTG, conventional training group

The results obtained from repeated-measures analysis of variance and t-tests are presented in detail in Table 2 and graphically illustrated in Fig. 2 (Fig. 2). A significant temporal effect was observed for all outcome measures across all three groups (Table 2).

At baseline, there were no significant differences between groups in FAAM-daily living activities (FAAM-DLA), FAAM-sport (FAAM-S), CAIT, FLT, BESS, YBT-A, YBT-PL, YBT-PM, RT, or SHT scores (*p* > 0.05) (Table 2).

Post-test and follow-up FAAM-DLA scores were significantly higher in both the DNSG and BTG compared to the CTG (F = 979.6; *p* = 0.00; η² =0.97). A significant time effect was also observed across all groups (F = 115.3; *p* = 0.00; η²=0.87), and post hoc comparisons revealed that DNSG and BTG had significantly greater improvements than CTG at both post-test and follow-up. Similarly, FAAM-S scores were significantly higher in DNSG and BTG than in CTG at both post-test and follow-up (F = 810.3; *p* = 0.00; η²=0.96). A significant time effect was noted across all groups (F = 65.0; *p* = 0.00; η²=0.80), with DNSG and BTG showing superior improvements over time compared to CTG. While no significant group differences in CAIT scores were observed at post-test or follow-up (F = 1922.4; *p* = 0.00; η²=0.98), there was a significant main effect of time (F = 48.7; *p* = 0.00; η²=0.75). In all groups, post-test values were higher than follow-up scores, and both were greater than baseline. (Table 2)

In the post-test and follow-up assessments, FLT scores were significantly higher in DNSG compared to both BTG and CTG, while BTG also outperformed CTG (F = 696.5; *p* = 0.00; η² = 0.95). A significant time effect was observed across all groups (F = 7.0; *p* = 0.00; η² = 0.30); however, follow-up scores were higher than post-test scores, and both were lower than pre-test values in all groups. (Table 2)

For BESS scores, pre-test values were comparable across groups. In post-test and follow-up, DNSG and BTG showed significantly lower total error scores than CTG (F = 15.8; *p* = 0.00; η²=0.49), indicating better static balance. A significant time effect was found for all groups (F = 721.2; *p* = 0.00; η²=0.96), with post-test scores showing the most improvement, followed by slight increases at follow-up. Overall, all groups improved over time, but CTG consistently showed higher error scores than the intervention groups. Pre-test values for YBT-A and YBT-PL were similar between groups. At both post-test and follow-up, DNSG and BTG had significantly higher reach distances than CTG (YBT-A: F = 3033.2; *p* = 0.00; η²=0.99; YBT-PL: F = 6384.9; *p* = 0.00; η²=0.99). A strong time effect was observed (YBT-A: F = 93.3; *p* < 0.001; η²=0.85; YBT-PL: F = 265.9; *p* < 0.001; η²=0.94), with post-test scores higher than follow-up, and both greater than baseline. Similarly, pre-test YBT-PM values were not significantly different across groups. DNSG and BTG outperformed CTG at post-test (F = 4540.6; *p* = 0.00; η²=0.99), though no group differences were found at follow-up. A significant time effect was present (F = 184.7; *p* = 0.00; η²=0.92), with all groups improving over time, but showing slight declines from post-test to follow-up. (Table 2)

At post-test and follow-up, BTG demonstrated significantly better BlazePod™ RT scores compared to CTG (F = 1298.03; *p* < 0.001; η² = 0.975). A significant time effect was also observed across all groups (F = 46.96; *p* < 0.001; η² = 0.74). While post-test values were the highest, follow-up scores remained improved relative to baseline. Similarly, DNSG and BTG outperformed CTG in SHT scores at both post-test and follow-up (F = 633.95; *p* < 0.001; η² = 0.95). A significant effect of time was found (F = 27.06; *p* = 0.01; η² = 0.60), with post-test scores being superior to follow-up, and both higher than pre-test values. (Table 2)

**Table 2:** Comparison of pre-test, post-test and follow-up test findings of evaluation parameters across time and groups

Time	Groups			Group Comparison	Group	Group* Time		Time Comparison
	DNSG^1^	BTG^2^	CTG^3^					
	X̅±SD	X̅±SD	X̅±SD					
**FAAM-DLA (%)**								
**Pre** ^**a**^	71.09 ± 9.33	75.40 ± 7.86	71.36 ± 7.75	ND	F:979.6; *p* < 0.001; η2:0.97	F:115.0; *p* < 0.001; η2:0.87		a < b,c and c < b
**Post** ^**b**^	90.19 ± 9.37	86.80 ± 8.88	76.90 ± 7.95	3 < 1,2				
**Follow-up** ^**c**^	84.28 ± 8.88	84.41 ± 8.60	73.70 ± 8.08	3 < 1,2				
**FAAM-Sport (%)**								
**Pre** ^**a**^	64.86 ± 7.71	67.21 ± 7.57	65.70 ± 7.09	ND	F:810.3; *p* < 0.001; η2:0.96	F:65.0; *p* < 0.001; η2:0.80		a < b,c and c < b
**Post** ^**b**^	81.61 ± 9.08	83.74 ± 9.61	71.95 ± 7.28	3 < 1,2				
**Follow-up** ^**c**^	78.38 ± 8.96	80.13 ± 9.49	68.76 ± 7.13	3 < 1,2				
**CAIT**								
**Pre** ^**a**^	15.46 ± 2.99	16.92 ± 3.48	16.27 ± 3.95	ND	F:1922.4; *p* < 0.001; η2:0.98	F:48.7; *p* < 0.001; η2:0.75		a < b,c and c < b
**Post** ^**b**^	24.69 ± 3.30	24.50 ± 3.85	21.73 ± 5.02	ND				
**Follow-up** ^**c**^	22.92 ± 2.99	22.67 ± 3.80	20.09 ± 4.68	ND				
**FLT**								
**Pre** ^**a**^	7.23 ± 1.30	7.83 ± 1.11	8.36 ± 1.36	ND	F:696.5; *p* < 0.001; η2:0.95	F:4.6; *p* < 0.001; η2:0.22		a > b,c and c > b
**Post** ^**b**^	1.85 ± 0.55	2.75 ± 0.62	4.00 ± 0.89	1 < 2,3 and 2 < 3				
**Follow-up** ^**c**^	3.92 ± 0.95	5.17 ± 0.94	6.36 ± 1.12	1 < 2,3 and 2 < 3				
**BESS-Total**								
**Pre** ^**a**^	19.85 ± 2.58	20.67 ± 2.57	20.45 ± 2.84	ND	F:721.2; *p* < 0.001; η2:0.96	F:15.8; *p* < 0.001; η2:0.49		a > b,c and c > b
**Post** ^**b**^	7.77 ± 1.30	7.42 ± 1.24	12.27 ± 1.49	3 > 1,2				
**Follow-up** ^**c**^	13.54 ± 1.85	12.17 ± 2.25	16.55 ± 2.62	3 > 1,2				
**YBT-A**								
**Pre** ^**a**^	67.46 ± 3.20	67.00 ± 3.05	65.82 ± 4.79	ND	F:3033.3; *p* < 0.001; η2:0.99	F:93.34; *p* < 0.001; η2:0.92		a < b,c and c < b
**Post** ^**b**^	86.46 ± 3.62	83.83 ± 3.07	75.73 ± 5.57	3 < 1,2				
**Follow-up** ^**c**^	72.54 ± 3.20	72.67 ± 2.87	67.45 ± 4.97	3 < 1,2				
**YBT-PL**								
**Pre** ^**a**^	87.62 ± 2.53	87.92 ± 3.48	86.82 ± 3.92	ND	F:6384.9; *p* < 0.001; η2:0.99	F:265.9; *p* < 0.001; η2:0.94		a < b,c and c < b
**Post** ^**b**^	102.77 ± 2.98	99.83 ± 3.93	95.00 ± 4.49	3 < 1,2				
**Follow-up** ^**c**^	95.77 ± 2.74	96.25 ± 3.93	89.27 ± 4.17	3 < 1,2				
**YBT-PM**								
**Pre** ^**a**^	90.92 ± 2.33	91.17 ± 3.19	90.73 ± 3.82	ND	F:4540.6; *p* < 0.001; η2:0.99	F:184.7; *p* < 0.001; η2:0.92		a < b,c and b > c
**Post** ^**b**^	104.38 ± 2.57	101.17 ± 3.66	97.27 ± 4.10	3 < 1,2				
**Follow-up** ^**c**^	95.62 ± 2.22	95.42 ± 3.45	92.27 ± 4.10	ND				
**BlazePod™ RT**								
**Pre** ^**a**^	22.85 ± 3.02	23.00 ± 2.09	22.36 ± 2.29	ND	F:1298.03; *p* < 0.001; η2:0.975	F:46.96; *p* < 0.001; η2:0.74		a < b,c and b > c
**Post** ^**b**^	27.69 ± 3.57	29.67 ± 2.71	25.82 ± 2.40	2 > 3				
**Follow-up** ^**c**^	25.31 ± 3.33	26.75 ± 2.42	23.73 ± 2.28	2 > 3				
**SHT**								
**Pre** ^**a**^	10.71 ± 2.38	9.57 ± 2.50	9.21 ± 1.18	ND	F:633.95; *p* < 0.001; η2:0.95	F:27.06; **p:0.01**; η2:0.62		a < b,c and b > c
**Post** ^**b**^	5.45 ± 1.46	5.54 ± 1.71	7.17 ± 0.93	1,2 < 3				
**Follow-up** ^**c**^	6.92 ± 1.91	6.50 ± 2.10	8.15 ± 1.02	1,2 < 3				

F: Mixed design two-way dependent variance analysis (Mixed Repeated Measures ANOVA), X̅±SD: Mean ± standard deviation, a: Pre-test, b: Post-test, c: Following-up test, FAAM-DLA: Foot and ankle ability measure – daily living activities, FAAM-Sport: Foot and ankle ability measure – sport, CAIT: Cumberland ankle ınstability tool, FLT: Foot lift test, BESS: Balance error scoring system, YBT: Y balance test, RT: Reaction time; SHT: Side-hop test, DNSG: Dynamic neuromuscular stabilization training group, BTG: Balance training group CTG: Conventional training group

No deaths, withdrawals due to harms, or adverse events were observed in any of the study groups throughout the intervention and follow-up periods. Consequently, no estimated effect sizes or confidence intervals could be calculated for harm outcomes. All interventions were considered safe and well-tolerated as no participant reported pain, discomfort, or adverse effects during or after the intervention sessions, and no protocol-related adverse events occurred during the study.

## Discussion

This study provides valuable evidence on the comparative effectiveness of three rehabilitation strategies as DNS, balance training, and conventional physiotherapy for managing CAI in amateur athletes. DNS and balance training demonstrated superior outcomes across neuromuscular control and functional performance parameters, clearly outperforming conventional physiotherapy. Notably, DNS yielded the greatest improvements in postural control and dynamic balance, while balance training led to the most pronounced gains in RT. Both DNS and balance training also produced comparable and significant improvements in functional ability (FAAM-DLA and FAAM-S), static balance (BESS), and functional performance (SHT), indicating that they offer distinct but complementary benefits that support their integration into individualized rehabilitation programs.

The superiority of DNS and balance training was particularly evident in self-reported functional outcomes, with large effect sizes observed for FAAM-ADL (η²=0.97) and FAAM-Sport (η²=0.96). These findings are consistent with previous literature supporting the efficacy of DNS in enhancing postural control, coordination, and sensorimotor integration [53, 54]. Similarly, although balance training protocols have demonstrated moderate improvements in functional outcomes (d = 0.39–0.44) [31], the structured and multisensory approach used in this study may explain the greater effect sizes observed. Dynamic programs incorporating hop-stabilization have also shown functional gains in athletic populations with CAI [32], reinforcing the value of task-specific neuromuscular interventions. In contrast, the conventional training group achieved only limited gains, likely due to the absence of integrated sensorimotor elements.

Although no statistically significant group differences were detected in CAIT scores, DNS and balance training still yielded large effect sizes (η²=0.98), suggesting clinically meaningful improvements in perceived ankle stability. Direct evidence linking CAI to core control deficits is limited [55]; however, emerging findings indicate that individuals with CAI may demonstrate proximal impairments, such as altered lumbopelvic stability and trunk muscle function. These factors, combined with DNS’s established ability to enhance core control in other populations such as stroke and healthy adults [56, 57], may partly explain the sensorimotor improvements observed in our DNS group. Similarly, balance training led to substantial perceived gains in stability, aligning with previous findings from Díaz et al. [57] and Nam et al. [58]. Overall, our results suggest that proprioceptive and neuromotor strategies can reduce perceived instability, even if self-reported measures like CAIT do not always reveal statistical group differences.

DNS training produced the most significant improvements in the FLT, with a large effect size (η²=0.95), reflecting enhanced postural control. These findings are in line with studies by Kang et al. [59], Yoon et al. [56], and Panse et al. [23], which demonstrate DNS-related gains in dynamic balance and proximal stabilization. Balance training also improved FLT performance, supported by previous research using balance boards and resistance band protocols [45, 60]. These results underscore the value of both interventions in improving neuromuscular control during single-leg stance and functional movements.

In terms of static balance, both DNS and balance training groups outperformed the conventional group, with large gains in BESS total scores (η²=0.96). Notably, balance training led to especially strong improvements on foam-surface tasks, single-leg (η²=0.94) and tandem stance (η²=0.91), surpassing previous short-term interventions [61, 62]. DNS also contributed to static balance through its effects on central stabilization, as demonstrated in populations such as older adults and those with Parkinson’s disease [63–65]. Together, these results suggest that DNS may improve postural control through central motor control mechanisms, while balance training strengthens sensory integration and peripheral stabilization, making them synergistic in restoring static balance in CAI.

Dynamic balance performance, assessed via the YBT, improved significantly in the DNS and balance training groups across all directions, with effect sizes ranging from 0.92 to 0.94. These results align with previous work by Mahdieh et al. [19], but with even stronger effect sizes, possibly due to the inclusion of vestibular components in our protocol. The limited gains in the conventional group further underscore the need for task-specific neuromotor interventions that challenge the vestibular and proprioceptive systems.

Visual RT also improved across all groups, with the balance training group showing the most substantial gains (η²=0.98). This supports previous findings that balance exercises can enhance neuromuscular responsiveness and visual-motor coordination [66], although DNS did not result in significantly greater improvements in this domain. Prior evidence indicates that DNS may influence RT when measured electromyographically [67], but visually cued motor responses may require longer or more intense protocols. These results suggest that balance training may offer more immediate benefits in reaction performance, whereas DNS effects may emerge more gradually.

Functional performance, as assessed by the SHT, improved significantly in DNS and balance training groups compared to the conventional group (η²=0.95). These improvements reflect gains in lower limb reactivity, coordination, and agility. Prior studies have reported similar effects of DNS on speed and endurance [68], and enhanced athletic capacity following balance training [45, 60], further validating the combined utility of these approaches for performance enhancement in CAI.

The observed outcomes are likely mediated by distinct but complementary neuromuscular mechanisms. DNS, based on developmental kinesiology, facilitates central stabilization by activating deep core musculature and regulating intra-abdominal pressure [23, 25], which supports proximal control and efficient load transfer. In contrast, balance training targets somatosensory, visual, and vestibular inputs that form part of peripheral sensory systems, stimulating proprioceptors and the vestibular apparatus to enhance reflexive stabilization and sensory reweighting [69–71]. These mechanisms likely account for the broad improvements seen across postural control, dynamic balance, and RT, reinforcing the value of interventions that engage both central motor and peripheral sensorial pathways.

The findings are also consistent with the theoretical frameworks guiding both interventions. DNS aligns with developmental motor control theory by reactivating subcortical patterns for trunk stabilization [72], while balance training reflects systems theory by integrating multisensory input for postural adaptation [73]. Together, the results support a dual-framework model of CAI rehabilitation, suggesting that future protocols should consider both central and peripheral deficits in neuromotor function.

The strengths of this study include its randomized controlled design, blinded outcome assessment, and use of both objective and subjective outcome measures. The inclusion of innovative tools such as BlazePod™, alongside structured, theory-based neuromuscular training, enhances the ecological validity and clinical relevance of the findings. The three-group design allowed a direct comparison between interventions primarily targeting central motor control mechanisms, namely DNS, and those focusing on peripheral sensory systems, namely balance training, as well as a conventional training approach, while the 12-week follow-up provided valuable short-term data. Additionally, the interventions’ alignment with well-established theoretical models strengthens their translational potential.

Nonetheless, several limitations should be acknowledged. The inclusion of athletes from various sports disciplines may limit generalizability of findings to sport-specific populations. As the sample consisted exclusively of amateur athletes, the applicability of results to elite or sedentary individuals remains uncertain. The single-blind design introduces a risk of performance and assessment bias, as neither participants nor therapist were blinded. Non-affected legs were not assessed in the YBT; therefore, no direct leg-to-leg or side-to-side temporal comparisons could be made. This decision was made to minimize testing burden given the comprehensive nature of the assessment protocol. Additionally, psychosocial variables such as fear-avoidance beliefs, which may influence rehabilitation outcomes, were not assessed. Furthermore, ankle dorsiflexion range of motion was not measured, limiting the biomechanical interpretation of outcomes. These limitations should be considered when interpreting the study findings.

Future research should include longer follow-up periods to assess the durability of effects and consider sport- and age-specific adaptations. The inclusion of psychological and social domains would also contribute to a more holistic understanding of recovery in CAI. Comparative trials across elite, recreational, and sedentary populations are warranted to refine population-specific rehabilitation protocols.

This study highlights the clinical value of both DNS and balance training in the rehabilitation of athletes with CAI. DNS may be particularly beneficial for enhancing proximal stability and motor control, whereas balance training supports sensory reweighting and reactive postural strategies under unstable conditions. The observed improvements may strengthen the view that CAI involves deficits in both central motor control mechanisms and peripheral sensory systems. These findings may support the importance of integrating and individualizing targeted motor control and sensory integration strategies into rehabilitation frameworks to optimize outcomes and reduce the risk of reinjury.

## Conclusion

In conclusion, this study found improvements in all groups of amateur athletes with CAI, with DNS and balance training showing greater benefits than conventional physiotherapy. These findings support the effectiveness of DNS and balance training in enhancing neuromuscular control, postural stability, and functional performance, highlighting the value of incorporating targeted motor control and sensory integration strategies into rehabilitation programs. However, the relatively short follow-up period and limited representation of different sport disciplines may restrict the broader applicability of the findings. Future research should explore long-term outcomes and the investigate the differential impacts of these interventions across athletic populations and activity levels. Despite these limitations, the present findings have meaningful implications for clinical rehabilitation and sports science, highlighting the need for multifaceted, evidence-based approaches in managing CAI.

## Supplementary Information

Below is the link to the electronic supplementary material.

**Supplementary Material 1**: **Appendix A.** Exercise protocol for the dynamic neuromuscular stabilization training group. **Appendix B.** Exercise protocol for the balance training group. **Appendix C.** Exercise protocol for the conventional training group.

**Supplementary Material 2**

## Data Availability

The datasets generated and/or analyzed during the current study are available from the corresponding author upon reasonable request. De-identified participant data, intervention materials, and analysis code can be shared following approval.De-identified participant data, data dictionary and analytical code used to process the data are shared. Materials related to the intervention, including exercise protocols, progression charts, and instructional documents are shared. All data and materials will be available upon reasonable request from the corresponding author. Interested researchers should contact the corresponding author via email with a brief explanation of the research purpose. Data sharing will be granted after approval by the principal investigator and may require a data use agreement to ensure ethical standards are maintained.

## Declarations

### Protocol and statistical analysis plan

Protocol and statistical analysis plans are available upon request from the corresponding author. The statistical analysis plan is also included in the Methods section of the manuscript and is available upon request.

### Data sharing

De-identified participant data, data dictionary and analytical code used to process the data are shared. Materials related to the intervention, including exercise protocols, progression charts, and instructional documents are shared. All data and materials will be available upon reasonable request from the corresponding author. Interested researchers should contact the corresponding author via email with a brief explanation of the research purpose. Data sharing will be granted after approval by the principal investigator and may require a data use agreement to ensure ethical standards are maintained.

### Use of generative AI and AI-enabled technologies in the writing process

No generative AI or large language models were used in the drafting or editing of this manuscript.

## Abbreviations

BMI: Body mass index
BESS: Balance error scoring system
BTG: Balance training group
CAI: Chronic ankle instability
CAIT: Cumberland ankle instability tool
CONSORT: Consolidated standards of reporting trials
CTG: Conventional training group
DNS: Dynamic neuromuscular stabilization
DNSG: Dynamic neuromuscular stabilization group
FAAM-DLA: Foot and ankle ability measurement - activities of daily living subscale
FAAM-S: Foot and ankle ability measurement - sports subscale
FLT: Foot lift test
RT: Reaction time
SD: Standard deviation
SHT: Side hopTest
YBT: Y Balance test
YBT-A: Y Balance test – anterior
YBT-PL: Y Balance test – posterolateral
YBT-PM: Y Balance test – posteromedial
