# Supplementary material for: Dynamic neuromuscular stabilization, balance, and conventional training for chronic ankle instability in amateur athletes: a randomised controlled trial
Source: BMC Sports Sci Med Rehabil. 2025 Oct 1;17:286. doi: 10.1186/s13102-025-01319-8 (PMC12486776; doi:10.1186/s13102-025-01319-8)
Supplement: Supplementary file 1 — Supplementary Material 1: Appendix A. Exercise protocol for the dynamic neuromuscular stabilization training group. Appendix B. Exercise protocol for the balance training group. Appendix C. Exercise protocol for the conventional training group. [file 13102_2025_1319_MOESM1_ESM.docx]

**Appendix A.** Exercise protocol for the dynamic neuromuscular stabilization training group

| **Exercise** | **Definition** | **Initial Repetitions/ Duration** | **Progression** | **Photographs** |
| --- | --- | --- | --- | --- |
| **Baby Rock** | The participant lies on their back, with the head, chest, spine and pelvis in a neutral position, the neck neutral, the vertebrae in contact with the surface, and the hips and knees bent to 90°, supported by the calves. | 5 repetitions, 30 seconds each | 5 repetitions, 60 seconds each | 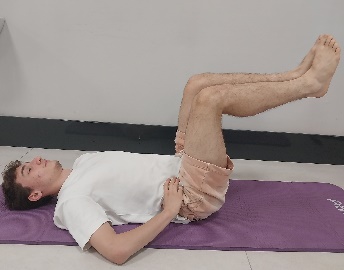 |
| **Prone Position** | The participant lies face down with support under the head, elbows positioned in front of the shoulders at ear level, shoulders wide without retraction, and the trunk supported at the symphysis or ASIS level, with the head slightly raised. | 5 repetitions, 30 seconds each | 5 repetitions, 60 seconds each | 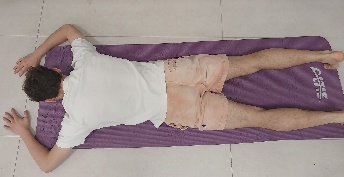 |
| **Bent Over Position** | a) Oblique Sitting, Elbow Support Position: The participant sits sideways, supported by forearm and hip, while maintaining a straight spine, neck and head.  b) Oblique Sitting, Hand Support Position: The participant is positioned in a hand-supported, inclined sitting position, with the lower leg semi-bent, the upper leg supported by the foot, and the spine maintained in a straight alignment. | 5 repetitions, 30 seconds each | 5 repetitions, 60 seconds each | a) 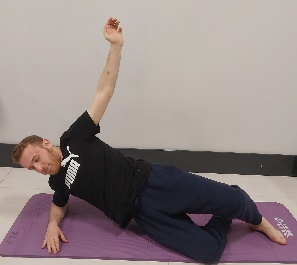  b) 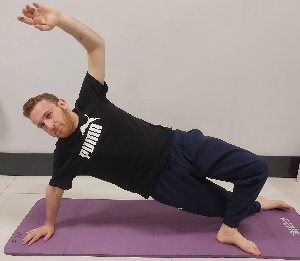 |
| **Tripod Position** | The participant is in a tripod position with hands shoulder-width apart and one foot supported at the same level as ipsilateral hand level. | 5 repetitions, 30 seconds each | 5 repetitions, 60 seconds each | 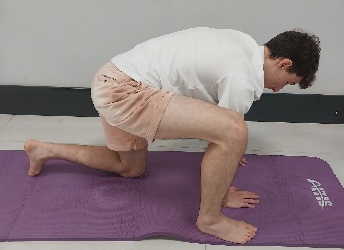 |
| **Hanging (Midstance/Push-Up)** | a) Midstance: One leg is on the stretcher, hip abducted and slightly extended and knee flexed. The other leg fully pressed to the ground with knee slightly flexed, and the spine remains straight. One arm is placed on the stretcher, while the other arm remains relaxed.  b) Push-up: The participant is positioned standing with arms shoulder-width apart, leaning on a piece of furniture. One leg is placed on the stretcher in a flexed position, while the other leg is extended backward, with the metatarsals of the foot placed on the yoga block, toes facing forward and heel turned outward. | 5 repetitions, 30 seconds each | 5 repetitions, 60 seconds each | a) 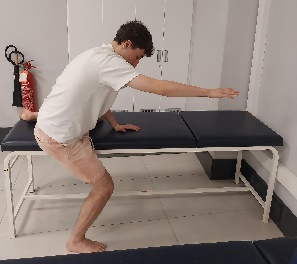  b) 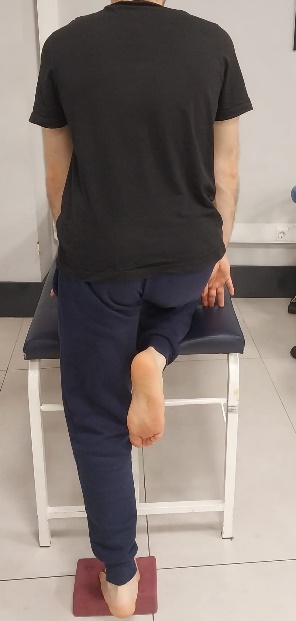 |
| **High Kneeling Position** | The participant is positioned with their arms resting on a piece of furniture, maintaining a spine straight. One leg is in kneeling position, while the other leg is placed in front, with the knee and hip at 90 degrees of flexion. The knee on the front leg is aligned so that it does not extend beyond the level of the big toe, and the toes are pointed straight ahead. | 5 repetitions, 30 seconds each | 5 repetitions, 60 seconds each | 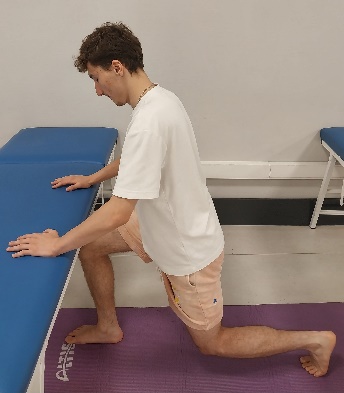 |
| **Bear Position** | The participant is positioned on the hands and feet, ensuring equal load distribution across the thenar and hypothenar aspects of the hands, with the shoulders aligned with the hands. The feet are supported by the forefeet or entire soles, the knees and hips are slightly bent, and the pelvis is elevated above the head. The spine is extended, maintaining a neutral position without any flexion or hyperextension. | 5 repetitions, 30 seconds each | 5 repetitions, 60 seconds each | 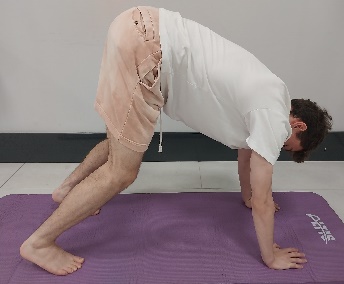 |
| **Squat Position** | The participant stands with their feet positioned at hip-width, keeping the spine, chest, and pelvis in a neutral alignment. | 5 repetitions, 30 seconds each | 5 repetitions, 60 seconds each | 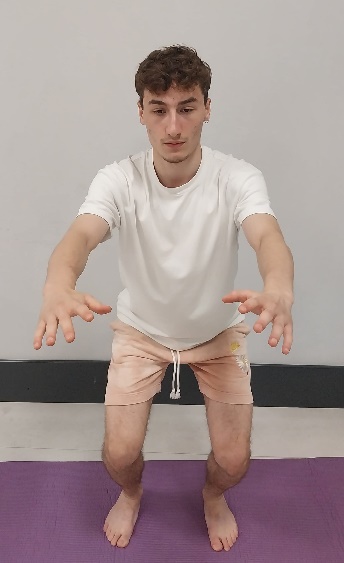 |

**Appendix B.** Exercise protocol for the balance training group

| **Exercise** | **Definition** | **Initial Repetitions/ Duration** | **Progression** | **Photographs** |
| --- | --- | --- | --- | --- |
| **Single-Leg Stance Exercise** | Single leg stance exercise is performed on a firm surface, balance pad, balance disc, balance board and BOSU. The patient maintains balance by standing upright on one leg and the exercise is performed with or without visual feedback. | 3 sets, 45 seconds | 3 sets, 60 seconds | 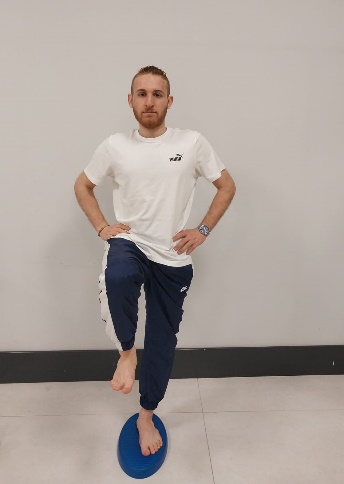 |
| **Ball Catching Exercise** | The exercise is performed in double-leg and tandem stances on a firm surface or balance pad. Also, a single-leg stance is performed on a firm surface, balance pad or balance disc. During the exercise, the patient is asked to follow the movement of the ball with his eyes and focus his attention on the ball's movement. | 1 set, 10 repetitions | 3 sets of 15 repetitions | 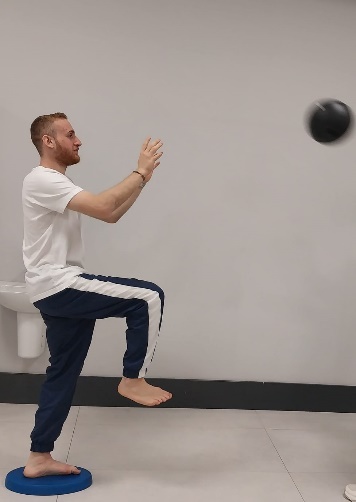 |
| **Forward-Backward and Side-to-Side Walking** | Head movements are performed on a firm surface or exercise mat while walking forward, backward or sideways, with the head moving vertically and laterally, while focusing on the ball. | 1 set, 10 repetitions | 3 sets of 15 repetitions | 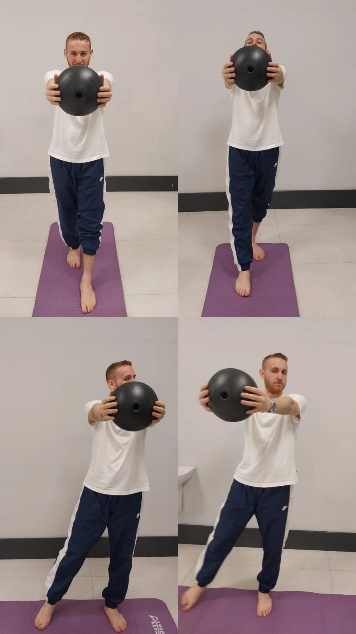 |
| **Perturbation Training** | The exercise is performed in a single-leg stance on a firm surface, balance pad, or BOSU ball. The patient is instructed to maintain balance against balls thrown from the front or pass them. | 1 set, 10 repetitions | 3 sets of 15 repetitions | 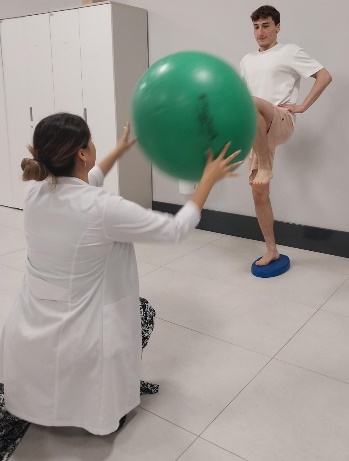 |
| **Hopping Stabilization Training** | Hopping exercises are performed on a firm surface, artificial turf and exercise mat. The participant performs hopping movements in the form of a square, zigzag, figure 8, straight line, and side to side jumps, while bouncing a size 4 or 5 soccer ball to maintain balance. | 1 set, 10 repetitions | 3 sets of 15 repetitions | 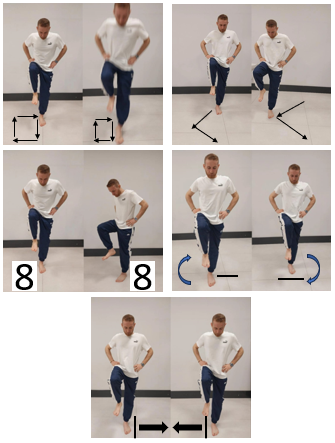 |
| **Hip Strengthening Exercises with a Pilates Band on Foam Surface** | The exercise is performed with the affected foot positioned on a firm surface, balance pad, balance disc, or BOSU ball. While standing on the affected foot, the participant performs hip flexion, extension, adduction, and abduction movements with the unaffected leg. | 1 set, 10 repetitions | 3 sets of 15 repetitions | 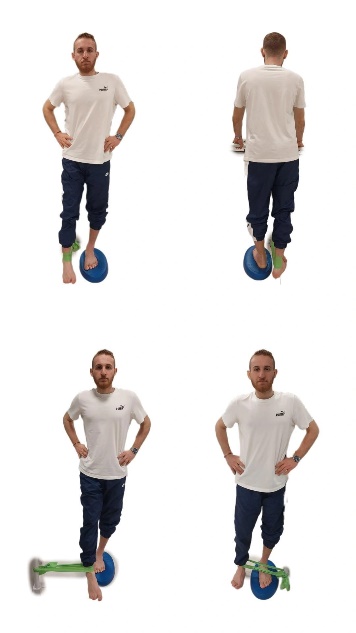 |
| **Lunge Exercise on Foam Surface** | The exercise is performed with the affected foot positioned on a balance pad in front and the unaffected leg placed behind on a balance pad or BOSU ball. The patient performs the movement while holding a ruler or weight in their hand and focuses their eyes on the object, which moves forward and backward. | 1 set, 10 repetitions | 3 sets of 15 repetitions | 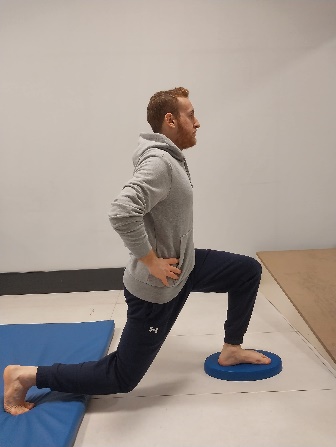 |

**Appendix C.** Exercise protocol for the conventional training group

| **Exercise** | **Definition** | **Initial Repetitions/ Duration** | **Progression** | **Photographs** |
| --- | --- | --- | --- | --- |
| **Warm-Up** | Stretching exercises (eccentric muscles of the toes, plantar fascia, hamstring, Achilles tendon, piriformis, gastrocnemius) | 1 set, 10 repetitions | 3 sets of 15 repetitions | 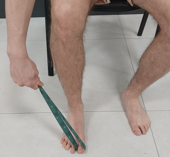  (Eccentric muscles of the toes) |
| **Joint Range of Motion** | Alphabet exercises | 1 set, 10 repetitions | 3 sets of 15 repetitions | 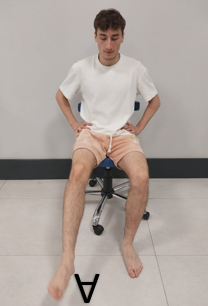 |
| **Strengthening** | a) Ankle strengthening exercises (isometric and isotonic)  b) Towel curl  c) Marble pickup | 1 set, 10 repetitions | 3 sets of 15 repetitions | a) 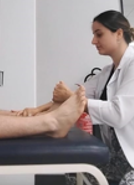 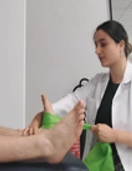  b) 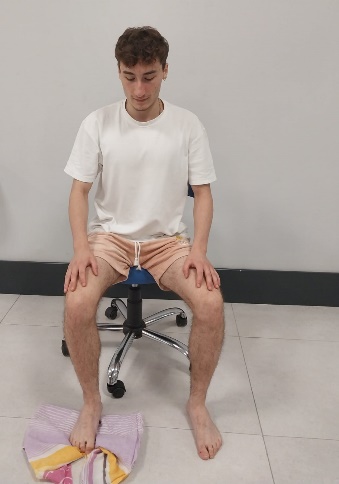  c) 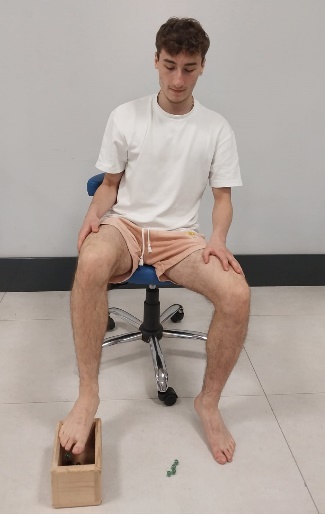 |
| **Postural Control** | a) Toe lifts  b) Walking on heels and toes  c) Ankle circles on a balance board | 1 set, 10 repetitions | 3 sets of 15 repetitions | a) 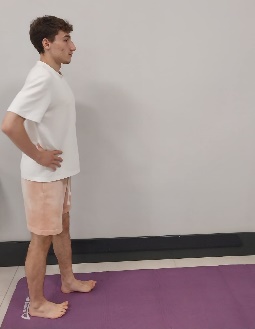  b) 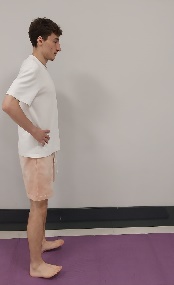 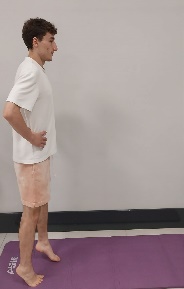  c) 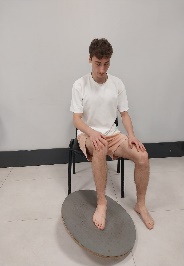 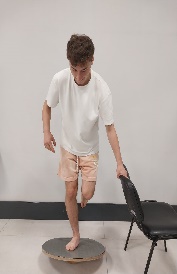 |
| **Cool-Down** | Submaximal running at an intensity between 30-40% of maximum heart rate | 1 set, 10 repetitions | 3 sets of 15 repetitions |  |
