# Supplementary material for: Dynamic neuromuscular stabilization, balance, and conventional training for chronic ankle instability in amateur athletes: a randomised controlled trial
Source: BMC Sports Sci Med Rehabil. 2025 Oct 1;17:286. doi: 10.1186/s13102-025-01319-8 (PMC12486776; doi:10.1186/s13102-025-01319-8)
Supplement: Supplementary file 2 — Supplementary Material 2 [file 13102_2025_1319_MOESM2_ESM.docx]

**CONSORT 2025 Checklist of Information to Include When Reporting a Randomised Trial**

| **Section/Topic** | **Item No.** | **Checklist Item** | **Reported on Page No.** |
| --- | --- | --- | --- |
| **Title and Abstract** | 1a | Identification as a randomized trial in the title | 1 |
|  | 1b | Structured summary of trial design, methods, results, and conclusions | 2,3 |
| **Trial Registration** | 2 | Registration number and registry name | 3,37,38 |
| **Protocol and SAP** | 3 | Access to full trial protocol and statistical analysis plan | 35 |
| **Data Sharing** | 4 | Data availability statement (how and where data can be accessed) | 36,37 |
| **Funding and Conflicts of Interest** | 5 | Sources of funding and roles of funders; conflicts of interest | 36 |
| **Background and Rationale** | 6 | Scientific background and explanation of rationale | 4-7 |
| **Objectives** | 7 | Specific objectives or hypotheses | 7 |
| **Patient and Public Involvement** | 8 | Description of any patient or public involvement in the design, conduct, or reporting of the trial | 7 |
| **Trial Design** | 9 | Description of trial design (e.g., parallel, factorial), including allocation ratio | 7 |
| **Changes to Trial Protocol** | 10 | Important changes to methods after trial commencement, with reasons | NA |
| **Trial Setting** | 11 | Settings and locations where data were collected | 8 |
| **Eligibility Criteria** | 12 | Eligibility criteria for participants | 8-10 |
| **Interventions** | 13 | Details of the interventions for each group, including how and when they were actually administered | 10,11,54,55,56 |
| **Outcomes** | 14 | Completely defined pre-specified primary and secondary outcome measures, including how and when they were assessed | 12-15 |
| **Harms (Description)** | 15 | All important harms or unintended effects in each group | 15,16 |
| **Sample Size** | 16 | How sample size was determined | 16,17 |
| **Sequence Generation** | 17 | Method used to generate the random allocation sequence | 17,18 |
| **Allocation Concealment Mechanism** | 18 | Mechanism used to implement the random allocation sequence and conceal until assignment | 17,18 |
| **Randomisation Implementation** | 19 | Who generated the sequence, enrolled participants, and assigned them | 17,18 |
| **Blinding** | 20 | If done, who was blinded and how | 18 |
| **Statistical Methods** | 21 | Statistical methods used to compare groups for primary and secondary outcomes | 18-20 |
| **Participant Flow (Diagram Recommended)** | 22 | Numbers randomly assigned, received treatment, analysed; losses and exclusions with reasons | 20,52 |
| **Recruitment** | 23 | Dates defining recruitment and follow-up periods | 21 |
| **Intervention Delivery** | 24 | Extent to which intervention was delivered as planned (fidelity) | 11 |
| **Baseline Data** | 25 | Table showing baseline demographic and clinical characteristics for each group | 21,22 |
| **Numbers Analysed, Outcomes, Estimation** | 26 | Number of participants in each analysis, outcomes, effect size, confidence intervals | 24,25,26 |
| **Harms (Results)** | 27 | Detailed reporting of adverse effects and harms results | 26 |
| **Ancillary Analyses** | 28 | Results of any other analyses performed (e.g., subgroup, adjusted) | NA |
| **Interpretation** | 29 | Interpretation of results considering limitations and context | 26-33 |
| **Limitations** | 30 | Trial limitations, sources of bias, imprecision, generalisability | 32 |
